# Investigation of Adaptive Optics Imaging Biomarkers for Detecting Pathological Changes of the Cone Mosaic in Patients with Type 1 Diabetes Mellitus

**DOI:** 10.1371/journal.pone.0151380

**Published:** 2016-03-10

**Authors:** Marco Lombardo, Mariacristina Parravano, Sebastiano Serrao, Lucia Ziccardi, Daniela Giannini, Giuseppe Lombardo

**Affiliations:** 1 Fondazione G.B. Bietti IRCCS, Via Livenza 3, 00198, Rome, Italy; 2 Consiglio Nazionale delle Ricerche, Istituto per i Processi Chimico-Fisici (CNR-IPCF), Viale Stagno D’Alcontres 37, 98158, Messina, Italy; 3 Vision Engineering Italy srl, Via Adda 7, 00198, Rome, Italy; Justus-Liebig-University Giessen, GERMANY

## Abstract

**Purpose:**

To investigate a set of adaptive optics (AO) imaging biomarkers for the assessment of changes of the cone mosaic spatial arrangement in patients with type 1 diabetes mellitus (DM1).

**Methods:**

16 patients with ≥20/20 visual acuity and a diagnosis of DM1 in the past 8 years to 37 years and 20 age-matched healthy volunteers were recruited in this study. Cone density, cone spacing and Voronoi diagrams were calculated on 160x160 μm images of the cone mosaic acquired with an AO flood illumination retinal camera at 1.5 degrees eccentricity from the fovea along all retinal meridians. From the cone spacing measures and Voronoi diagrams, the linear dispersion index (*LDi*) and the heterogeneity packing index (*HPi*) were computed respectively. Logistic regression analysis was conducted to discriminate DM1 patients without diabetic retinopathy from controls using the cone metrics as predictors.

**Results:**

Of the 16 DM1 patients, eight had no signs of diabetic retinopathy (noDR) and eight had mild nonproliferative diabetic retinopathy (NPDR) on fundoscopy. On average, cone density, *LDi* and *HPi* values were significantly different (P<0.05) between noDR or NPDR eyes and controls, with these differences increasing with duration of diabetes. However, each cone metric alone was not sufficiently sensitive to discriminate entirely between membership of noDR cases and controls. The complementary use of all the three cone metrics in the logistic regression model gained 100% accuracy to identify noDR cases with respect to controls.

**Conclusion:**

The present set of AO imaging biomarkers identified reliably abnormalities in the spatial arrangement of the parafoveal cones in DM1 patients, even when no signs of diabetic retinopathy were seen on fundoscopy.

## Introduction

Diabetic retinopathy (DR) is a chronic progressive sight-threatening disease of the retinal microvasculature and neuronal cells associated with prolonged hyperglycaemia. It represents one of the leading causes of visual impairment among adults in the western world and its incidence is increasing in other parts of the world, primarily India and China. People with diabetes mellitus are at higher risk of vision loss than the general population; the prevalence of this complication relates to the type and duration of diabetes.[1–3]

Early diagnosis of DR is required to preserve vision and avoid serious complications. Currently, diagnosis of DR is made when the damage has already happened at a macroscopic scale, due to limits of current functional and imaging instruments to evaluate structural impairments of cellular components of the neuro-retinal tissue. As innovative optical technologies are at disposal of clinicians, new approaches of early detection of pathological tissue changes can emerge. Adaptive optics (AO) retinal imaging has been shown to resolve non-invasively alterations of the photoreceptor mosaic in patients with diabetes mellitus.[4] In previous work, cone photoreceptor involvement in a cohort of eleven adult patients with a history of type 1 diabetes mellitus (DM1) in the past 9 years to 21 years has been examined and the decreased cone density within the central retina has been correlated with increasing duration of diabetes, the presence of diabetic retinopathy on fundoscopy and poor glycometabolic control.[4] However, several studies have demonstrated moderate to high variability within cone density even within the healthy population, making it difficult to detect small deviations from normal in controlled comparative studies.[5] Overall, cone density alone appears to be intrinsically unable to provide valuable information on the early pathological changes of the cone mosaic in patients with type 1 diabetes mellitus.

Previous work has shown that cone spacing analysis can be more sensitive and less prone to errors than density analysis when tracking disease progression or response to treatment in eyes with retinal degeneration.[6] In addition, investigation of the packing arrangement of cones has revealed significantly disrupted regularity of the photoreceptor mosaic in individuals with congenital tritan color vision deficiency and cone density within normal limits.[7]

In the present work, we explored a set of AO imaging biomarkers for detecting abnormalities in the cone density and non-random pattern of the parafoveal photoreceptor mosaic. The use of metrics for describing the cell spacing and packing arrangements in addition to density may improve the AO retinal imaging tools’ sensitivity and be valuable for detecting early pathological changes of the retinal mosaic in patients with type 1 diabetes.[8–13] Indeed, cone density gives information about the numerosity of points on local scale and does not provide any information about spatial organization of such points; the spacing of cones provides a measure of dispersion^14^ and the use of Voronoi diagrams provides a measure of spatial arrangement, since it is based on the arrangement of points in relation to one another.[15–17]

## Materials and Methods

All research procedures described in this work adhered to the tenets of Declaration of Helsinki. The study protocol was approved by the local ethics committee (Azienda Sanitaria Locale Roma A, Roma, Italy) and all subjects recruited gave written informed consent after a full explanation of the procedure.

Patients with a diagnosis of type 1 diabetes mellitus and age-matched healthy volunteers participated in this study. Inclusion criteria were an age >18 years old, diagnosis of diabetes from at least 4 years and definition of no or mild signs of nonproliferative diabetic retinopathy (NPDR) according to the ETDRS severity scale,[18,19] 20/20 or better Uncorrected or Best Corrected Visual Acuity (UCVA or BCVA). Exclusion criteria were astigmatism higher than 2.50 dioptres, the presence or a history of maculopathy or any other ocular disease, including lens opacity, or previous eye surgery. Mild NPDR was defined as the presence of at least one microaneurysm and/or mild hemorrhages; no DR was defined as the absence of any of the above signs.[18,19] Voluntary healthy subjects were recruited in the study as controls. Control subjects had no history of systemic or ocular diseases and no previous eye surgery.

All subjects recruited in the present study underwent comprehensive ophthalmologic examination including retinal imaging using a flood-illumination AO retinal camera (*rtx1*, Imaging Eyes, Orsay, France), a *Spectralis* (Heidelberg Engineering GmbH, Heidelberg, Germany) and a colour fundus retinal camera (TRC-50 DX, Topcon Instr. Corp, Tokyo, Japan). In addition, all subjects had non-contact ocular biometry using the *IOL Master* (Carl Zeiss Meditec AG, Hennigsdorf, Germany).

### Acquisition and analysis of adaptive optics retinal images

**Fig 1:**
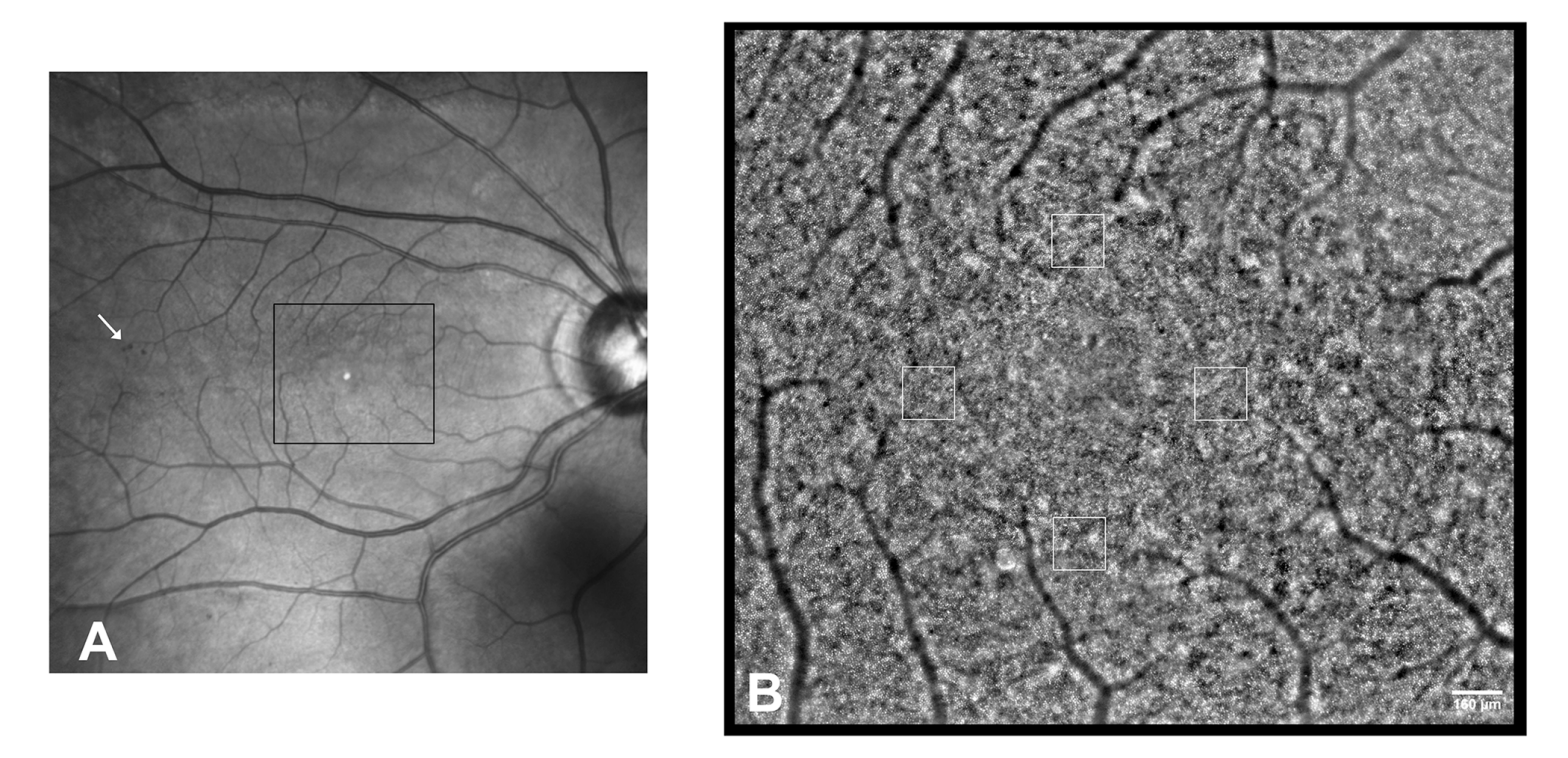
Adaptive optics montage of the central retina in the right eye of a patient with a diagnosis of type 1 diabetes mellitus in the past 37 years (DM1_P7). A) Wide field fundus image. The arrow highlights a cluster of spot hemorragies outside the region of interest. B) Adaptive optics montage of the central retina showing the photoreceptor mosaic (2.46x2.18 mm). Analysis of the cone mosaic was done in 160x160 μm sampling areas on four locations at 1.5 degrees eccentric from the fovea along all retinal meridians. Scale bar: 160 μm.

The imaging session was conducted after dilating the pupil with one drop of 1% tropicamide. During imaging, fixation was maintained by instructing the patient to fixate on the internal target of the instrument moved by the investigator. At each retinal location, a sequence of 40 frames was acquired by illuminating a retinal area subtending 4 degrees of visual angle in the right eye of each subject; images were acquired at several locations in the central retina covering an area of 5x4 degrees centered on the preferred locus of fixation (coordinates x = 0° and y = 0°) (Fig 1).

A proprietary program provided by the manufacturer was used to correct for distortions within frames of the raw image sequence and to register and frame-average in order to produce a final image with enhanced signal-to-noise ratio prior to further analysis.[8–10,20] The final images were montaged using the montage tool in i2k Retina Pro (DualAlign LLC, Clifton Park, NY, USA).

The corrected magnification factor (RMF_corr_) was calculated for each eye in order to correct for the differences in optical magnification and thus retinal image size between eyes, as previously described.[8–10,20–22] Cone labelling process was performed using an enhanced version of the algorithm implemented with the image processing toolbox in Matlab (The Mathworks Inc, Natick MA, USA), as previously reported.[8–10,23] The study protocol included analysis of 160x160 μm images of the cone mosaic, centered at 1.5 degrees eccentric from the foveal center, in each of the four directions (temporal, superior, nasal, inferior). The area was chosen in order to have a sampling window large enough to describe the mosaic arrangement of the parafoveal cones, as previously reported.[4,8–10] Attention was paid to place the sampling windows in areas devoid of large vessels or retinal haemorrhages. This approach ensured that no *undersampling effect* occurred in any sampling areas.[8–10] After automated cone identification, two expert investigators (ML and GL), who were masked to group assignment, reviewed each area and manually identified cones that they agreed to be missed or selected in error by the algorithm. The *x*,*y* coordinates of the cones were then stored in a text array and used to calculate cone density, spacing and packing arrangement.

Cone positions were converted into local densities by calculating their number per square millimeter (cones/mm^2^). The center-to-center cone distance (micrometers, μm) was calculated using a novel approach. For each retinal location, the cone spacing was determined by calculating the average distance from the center of each cone to the centers of six neighboring cones within an area of 12 pixels (9.6 μm) diameter. This area was chosen in order to limit the spacing analysis to a distance that is approximately twice the average cone spacing at 1.5 degrees eccentricity. The method is assumption free and provides estimates of both central tendency and variation. The standard deviation of cone spacing provides empirical measures of the actual spread in cell neighbour distances. In this study, we used the *linear dispersion index*, *LDi*, expressed as the ratio of *σ*_*spacing*_/*M*_*spacing*_, where σ_*spacing*_ is the standard deviation of the mean M_*spacing*_, to determine the local dispersion of cones in the given mosaic. The higher the *LDi*, the higher the spread in neighbour distances between cones. This metric was originally developed by Hirsch et al.[14,24] and termed *fractional spacing disorder*. Since there is no evidence of perfect regularity even in the normal cone mosaic, in this work we avoided to use the term “disorder”.[8]

The cone packing arrangement was analyzed using Voronoi diagrams.[8–10,15] The Voronoi tessellation was implemented by the *voronoi* Matlab function from the bidimensional coordinates of labelled cones. Each Voronoi cell was coded by a different colour corresponding to the number of their neighbouring cones: gray = tetragonal (4*n*) arrangement, yellow = pentagonal (5*n*) arrangement, green = hexagonal (6*n*) arrangement; blue = heptagonal (7*n*) arrangement and white = octagonal (8*n*) arrangement. The Voronoi regions lying at the bounds of each section were excluded from further analysis, creating a buffer zone = 2 NND in order to minimize the *boundary effect*.[10] In order to find possible alterations from the healthy spatial-distribution pattern of the cones,[25,26] we developed the *heterogeneity packing index*, *HPi* (percentage, %), which is calculated as 6*n*-(8*n*+4*n*) and represents the fractional increase in 4-sided and 8-sided non hexagonal Voronoi tiles with respect to 6-sided cells. The lower the *HPi*, the higher the deviation of the cone mosaic from normal arrangement.

### Statistics

Statistics were performed using the SPSS software (version 17.1; SPSS Inc., Chicago, IL, USA).

Sample size calculation (allocation ratio 1:3) was performed to detect a difference of 1500 cones/mm^2^ between the mean cone density for the NPDR and noDR groups and controls, at a significance level of 5% and a power of 80%, assuming a standard deviation of 1250 cones/mm^2^. For each subject, the cone density, *LDi* and *HPi* values were averaged from the superior, inferior, temporal and nasal locations in order to achieve a global value of the spatial distribution and organization of cones by sampling data of the parafoveal locations. This approach was chosen in order to define a standardized protocol to screen the integrity of the cone mosaic in patients. The normal data distribution was tested by using the one-sample Kolmogorov-Smirnov test; the one-way analysis of variance (ANOVA) test was then used to statistically compare the differences among groups; age was taken into account when comparing data. The 95% confidence level (95% Cl) was calculated as $\pm 1.96\sigma /\sqrt{n}$, where *n* is the number of observations. The Pearson correlation coefficient was calculated to correlate the changes of the cone metrics as a function of duration of diabetes or age.

In order to validate the complementary use of the present set of AO biomarkers to assess abnormalities of the parafoveal photoreceptor mosaic early in the course of DM1 in adults, the binary logistic regression model [27] was applied using the three cone metrics (density, *LDi* and *HPi*) as explanatory, independent, variables (i.e., predictors) to classify noDR cases and controls (i.e., the categorical dependent variables). Logistic regression does not need a linear relationship between the dependent and independent variables; however, it requires 1) the dependent variable to be binary and ordinal; 2) the independent variables to be linearly related to the log odds and 3) the model to have little or no multicollinearity (i.e., the independent variables should be independent from each other). The variance inflation factor (VIF), which assesses how much the variance of an estimated regression coefficient increases if the predictors are correlated, was used to assess multicollinearity. A VIF between 5 and 10 indicates high correlation among predictors and a VIF above 10 indicates that the regression coefficients are poorly estimated due to multicollinearity. In this study, the maximum threshold of VIF outcome was set at 5. Statistically significant differences were set at *P*<0.05 for all the tests performed.

## Results

### Participants

Thirty-six participants were recruited in this study. Sixteen were patients with type 1 diabetes mellitus and the other twenty were healthy volunteers (Table 1).

**Table 1:** Characteristics of the study population. Patients with type 1 diabetes mellitus were identified as having mild nonproliferative diabetic retinopathy (NPDR) or not (noDR) by fundoscopy.

Case	Age	Gender	Duration of diabetes	HBA1c	Diagnosis	BCVA (Snellen)
**DM1_P1**	37	F	21	7.2	NPDR	1.0
**DM1_P2**	35	F	17	6.1	NPDR	1.0
**DM1_P3**	32	M	16	8.2	NPDR	1.0
**DM1_P4**	35	F	10	6.8	NPDR	1.2
**DM1_P5**	50	F	13	7.2	NPDR	1.0
**DM1_P6**	48	M	11	7.9	NPDR	1.0
**DM1_P7**	55	F	37	7.0	NPDR	1.0
**DM1_P8**	51	M	18	8.0	NPDR	1.0
**DM1_P9**	35	F	9	6.3	noDR	1.2
**DM1_P10**	46	M	9	8.9	noDR	1.0
**DM1_P11**	39	M	12	7.0	noDR	1.2
**DM1_P12**	36	M	11	8.2	noDR	1.2
**DM1_P13**	30	M	8	7.6	noDR	1.2
**DM1_P14**	36	F	9	7.0	noDR	1.0
**DM1_P15**	47	M	12	8.0	noDR	1.2
**DM1_P16**	29	F	14	8.0	noDR	1.0
**C30**	29	F	-	-	Control	1.0
**C31**	27	F	-	-	Control	1.2
**C32**	28	F	-	-	Control	1.2
**C33**	34	M	-	-	Control	1.0
**C34**	28	M	-	-	Control	1.2
**C35**	29	F	-	-	Control	1.2
**C36**	40	F	-	-	Control	1.0
**C37**	39	F	-	-	Control	1.0
**C38**	28	F	-	-	Control	1.2
**C39**	42	M	-	-	Control	1.2
**C40**	27	F	-	-	Control	1.0
**C41**	47	M	-	-	Control	1.2
**C42**	30	F	-	-	Control	1.0
**C43**	36	M	-	-	Control	1.2
**C44**	39	F	-	-	Control	1.0
**C45**	33	M	-	-	Control	1.2
**C46**	56	M	-	-	Control	1.0
**C47**	44	F	-	-	Control	1.0
**C48**	52	F	-	-	Control	1.0
**C49**	38	F	-	-	Control	1.2

**HBA1c** = glycohemoglobin serum level; **BCVA** = Best Corrected Visual Acuity

Eight patients (5 males and 3 females; age range 29–47 years old; mean age: 37.0 ± 6.6 years) affected by DM1 in the past 8 years to 14 years (mean: 10.5 ± 2.1 years), had a diagnosis of noDR on fundoscopy. In these patients, the mean A1c form of glycohaemoglobin (HbA1c) level was 7.5 ± 0.8% (range: 6.3–8.9%). The other eight patients (3 males and 5 females; age range: 32–55 years old; mean age: 42.8 ± 9.0 years), who were affected by DM1 in the past 10 years to 37 years (mean: 17.9 ± 8.5 years), had a diagnosis of NPDR on fundoscopy. In these patients, the mean HbA1c level was 7.3 ± 0.7% (range: 6.1–8.2%). Twenty healthy subjects (7 males and 13 females; age range: 27–56 years; mean age: 36.4 ± 8.6 years) were recruited as controls (Table 1). No statistically significant differences were found between the mean age for the NPDR (P = 0.08) and noDR (P = 0.08) groups and controls.

The mean corrected magnification factor (RMF_corr_) was 0.276 ± 0.007 mm/deg, 0.284 ± 0.016 mm/deg and 0.280 ± 0.012 mm/deg in noDR eyes, NPDR eyes and controls respectively.

No macular edema was identified by SD-OCT imaging in any case.

### Cone metrics

The automated identification algorithm showed to be highly accurate to identify cones in AO images of the parafovea in DM1 patients and controls; the mean percentage of manually added cones ranged between 0.5% and 5.6%, with no differences between patients with type 1 diabetes (mean 1.4±1.1%) and controls (mean 1.5±1.1%).

On average, cone density was significantly lower in NPDR (26585 ± 1377 cones/mm^2^; P<0.001) and noDR (27855 ± 970 cones/mm^2^; P<0.001) eyes than controls (29452 ± 1484 cones/mm^2^). In controls, aging was not significantly correlated to decreased cone density (r = -0.40; P = 0.08); in DM1 patients, density decreased significantly with increasing duration of diabetes (r = -0.60; P<0.001). In NPDR eyes, the average paravofeal cone density was 11% lower than controls (P<0.001); in noDR eyes, it was 6% lower than controls (P = 0.01; Table 2).

**Table 2:** Average (±SD) values of the cone metrics calculated by sampling data of the four paravofeal locations at 1.5 degrees eccentric from the fovea in patients with type 1 diabetes mellitus (DM1) with (NPDR) or without (noDR) signs of diabetic retinopathy on fundoscopy and age-matched controls.

Case	Cone density (cones/mm^2^)	Linear Dipersion index (dimensionless unit)	Heterogeneity Packing index (%)
**DM1_P1**	26998	0.093	0.354
**DM1_P2**	25798	0.087	0.381
**DM1_P3**	29307	0.077	0.400
**DM1_P4**	24404	0.094	0.333
**DM1_P5**	26875	0.086	0.404
**DM1_P6**	26589	0.090	0.357
**DM1_P7**	26099	0.085	0.390
**DM1_P8**	26608	0.094	0.332
**DM1_P9**	28135	0.084	0.403
**DM1_P10**	27099	0.091	0.324
**DM1_P11**	25977	0.086	0.413
**DM1_P12**	28852	0.079	0.370
**DM1_P13**	28392	0.083	0.381
**DM1_P14**	28906	0.083	0.402
**DM1_P15**	27754	0.089	0.336
**DM1_P16**	27725	0.084	0.374
**C30**	30977	0.077	0.405
**C31**	28414	0.077	0.460
**C32**	30148	0.072	0.419
**C33**	30250	0.077	0.420
**C34**	28757	0.087	0.387
**C35**	29004	0.079	0.450
**C36**	31188	0.074	0.439
**C37**	29880	0.081	0.380
**C38**	32333	0.076	0.411
**C39**	30065	0.079	0.431
**C40**	29715	0.074	0.449
**C41**	25719	0.079	0.460
**C42**	30430	0.079	0.394
**C43**	28084	0.079	0.446
**C44**	28898	0.076	0.428
**C45**	29814	0.073	0.444
**C46**	28165	0.075	0.430
**C47**	30005	0.076	0.453
**C48**	27244	0.074	0.441
**C49**	29747	0.073	0.449
**NPDR (P1 –P8)**			
Mean	26585	0.088	0.369
SD	1377	0.006	0.029
95% Cl*	26054–27021	0.085–0.091	0.359–0.379
**noDR (P9 –P16)**			
Mean	27855	0.085	0.375
SD	970	0.004	0.032
95% Cl	27487–28142	0.083–0.087	0.364–0.387
**Controls**			
Mean	29452	0.077	0.431
SD	1484	0.004	0.024
95% Cl	29120–29784	0.076–0.079	0.424–0.435

* **Cl**: Confidence level

The average *LDi* was significantly higher in NPDR (0.088 ± 0.006; P<0.001) and noDR (0.085 ± 0.004; P<0.001) eyes than controls (0.077 ± 0.004). The *LDi* values increased with increasing duration of diabetes (r = 0.60; P<0.001). In NPDR eyes, the average parafoveal *LDi* was 13% higher than controls (P<0.001); in noDR eyes, it was 8% higher than controls (P = 0.04; Table 2).

**Fig 2:**
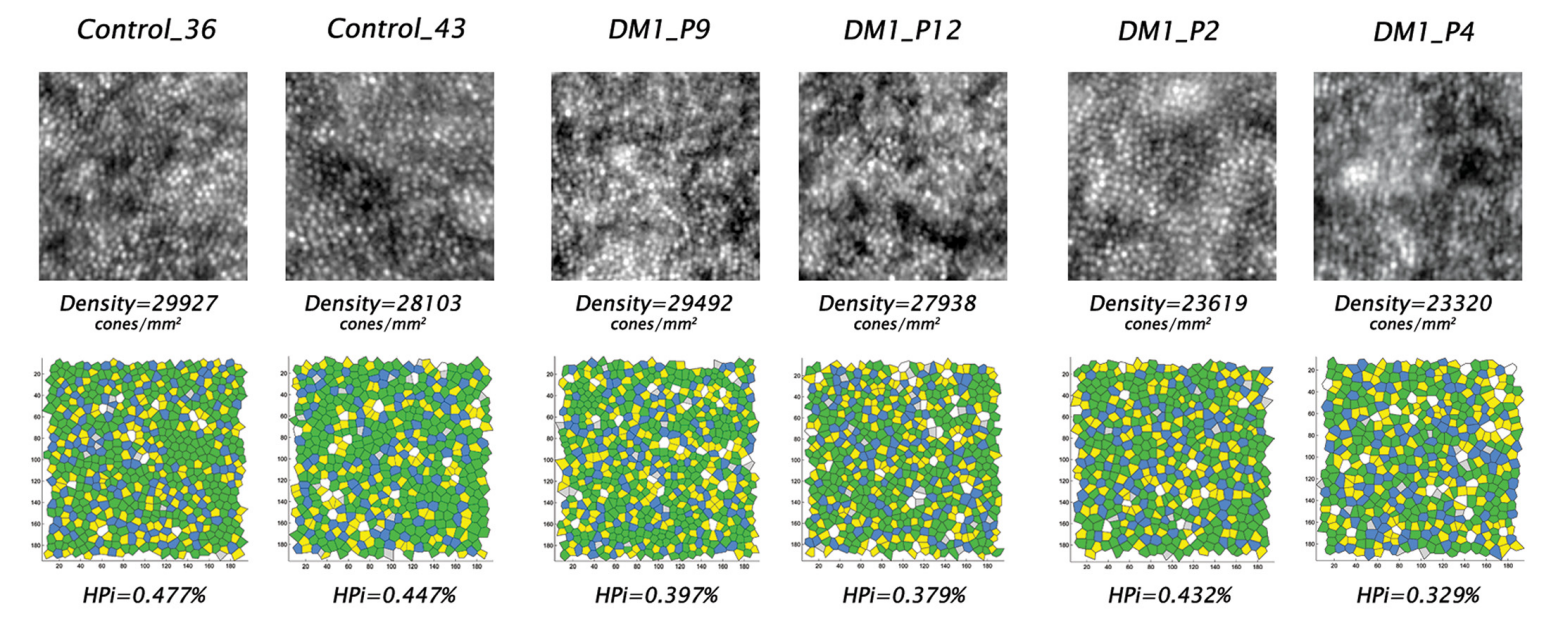
Adaptive optics images of the parafoveal cone mosaic and corresponding Voronoi diagrams in four patients with type 1 diabetes mellitus and two age-matched controls. Upper row) Adaptive optics images of the parafoveal cone mosaic (160x160 μm) and bottom row) corresponding Voronoi diagrams in six age-matched representative cases (colour maps are described under Methods). From the left to the right are shown the images collected at 1.5 degrees inferior from the fovea in Control_36, Control_43, DM1_P9, DM1_P12, DM1_P2 and DM1_P4 respectively. The corresponding data from this parafoveal location were as follows: in Control_36 (39 years old), cone density was 29927 cones/mm^2^ and percentages of 6*n* and 4*n*+8*n* were 51% and 3% respectively; in Control_43 (33 years old), cone density was 28103 cones/mm^2^ and percentages of 6*n* and 4*n*+8*n* were 51% and 6% respectively; in DM1_P9 (35 years old, duration of DM1: 9 years), density was 29492 cones/mm^2^, and percentages of 6*n* and 4*n*+8*n* were 46% and 6% respectively; in DM1_P12 (36 years old, duration of DM1: 11 years), density was 27938 cones/mm^2^, and percentages of 6*n* and 4*n*+8*n* were 45% and 7% respectively; in DM1_P2 (35 years old, duration of DM1: 17 years), density was 23619 cones/mm^2^, and percentages of 6*n* and 4*n*+8*n* were 47% and 5% respectively; in DM1_P4 (35 years old, duration of DM1: 10 years), density was 23320 cones/mm^2^, and percentages of 6*n* and 4*n*+8*n* were 41% and 8% respectively. The packing arrangement of the parafoveal cones in eyes with diabetes showed on average less hexagonal cones and more non-hexagonal cones, which were associated with average decreased cone density, in comparison with controls.

On average, the percentage of 6*n* Voronoi tiles was 7% higher (P<0.001) in controls than in DM1 patients (Fig 2). On the other hand, the percentage of non hexagonal arrangements were significantly higher in DM1 patients than controls: the mean percentage of 4*n* and 8*n* Voronoi tiles was 36% and 24% higher (P<0.001) than controls respectively; the mean percentage of 5*n* (P = 0.001) and 7*n* (P = 0.009) Voronoi tiles was 4%, higher in DM1 patients than controls. No differences (on average <1.1%) were found between NPDR and noDR eyes for each Voronoi tile. The average *HPi* was significantly lower in NPDR (0.369 ± 0.029; P<0.001) and noDR (0.375 ± 0.032; P<0.001) eyes than controls (0.431 ± 0.024); it decreased with increasing duration of diabetes (r = -0.54; P<0.001). In NPDR and noDR cases, the average parafoveal *HPi* values were 14% (P<0.001) and 13% (P<0.001) lower than controls respectively (Table 2).

### Logistic regression analysis

**Fig 3:**
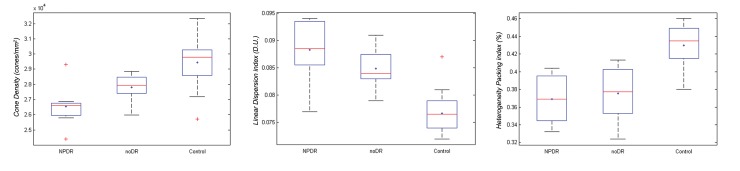
Box plots showing the distribution of values for cone density, linear dispersion index and heterogeneity packing index in patients with diabetes and controls. Parallel box plots showing the distribution of values for parafoveal cone density (left plot; cones/mm^2^), linear dispersion index (middle plot; *LDi*, D.U.: dimensionless unit) and heterogeneity packing index (right plot; *HPi*, %) in patients with (NPDR) and without (noDR) signs of diabetic retinopathy on fundoscopy and controls. The blue plus sign and the line in each box represent the mean and median scores respectively; the lower and upper edges of each box represent the 25^th^ and 75^th^ percentiles, respectively. The upper and lower adjacents (i.e., the bars) include all the extreme values up to the 99^th^ percentile. Red crosses represent outlier data points.

Although the cone metrics were statistically significantly different between the study and control groups, each of them alone was not sufficiently sensitive to discriminate entirely between membership of patients with diabetes and no signs of diabetic retinopathy on fundoscopy and controls (Fig 3). A logistic regression analysis was conducted to identify the specific parafoveal cone mosaic spatial organization in noDR eyes in comparison with controls using all the cone metrics as descriptors. In the present model, the VIF outcomes were <3.1 and the predictors were linearly related to the log odds, thus verifying both key assumptions of logistic regression.

By adding all the independent variables to the logistic regression model, it gained 100% accuracy to predict membership of eyes without signs of DR on fundoscopy and controls. Cox and Snell R^2^ index gained a value of 0.70, with Nagelkerke *R*^2^ = 1.00 (this parameter permits to estimate how much of the variability in the outcome variable can be explained by the model; 1.00 is the maximum value), indicating a strong relationship between prediction and grouping (S1 Fig). The *HPi* showed the best performance in the model; the scores were 13.48 (P<0.001), 11.87 (P = 0.001) and 6.79 (P = 0.009) for *HPi*, *LDi* and cone density respectively.

## Discussion

In this work, we have shown that the complementary use of density, spacing and packing arrangement of cones is valuable to detect early abnormalities of the parafoveal cone mosaic in adult patients with type 1 diabetes.

On average, cone density in NPDR cases (mean duration of diabetes: 18 years) was 11% lower than age-matched controls, consistent with the results of previous study on a different population of DM1 patients (10% lower cone density than controls; mean duration of diabetes: 14 years).[4] The cone density was 6% lower in noDR cases (mean duration of diabetes: 10 years) than controls. Although this difference was statistically significant, it cannot be considered clinically significant, due to the variability of cone density across the normal adult population. Thus, the present results confirm that cone density alone is unable to clearly identify the early pathological changes of the parafoveal cone mosaic in DM1 patients. Recently, Tan et al.[28] investigated the retinal periphery (7 degrees from the fovea) of 29 adolescents and young adults (10–25 years old) with diagnosis of DM1 at early age and no signs of DR on fundoscopy, showing no differences in cone density with age-matched controls. Direct comparison with the present and previous studies [4] cannot be made, since cone density has been evaluated in different retinal regions and the cohort of participants had different ages and duration of diabetes.

The complementary use of cone density, *LDi* and *HPi* in the logistic regression model gained 100% accuracy to identify membership of DM1 patients without signs of diabetic retinopathy on fundoscopy and controls. In noDR eyes, the *HPi* and *LDi* performed better than cone density to enhance the predictive power of the model.

In this study, cone spacing was calculated taking into account the six nearest neighbours to the given cone within a given area (i.e., twice the size of the cone at the given retinal location), thus minimizing the main limit of spacing measures that are based on the distribution of distances to nearest-neighbours.[8,14] Indeed, the main weakness of the nearest-neighbour distance (NND) is that it takes into account only the nearest of each cone’s known neighbours, regardless of its distance, and thus can be strongly influenced by very large NNDs of isolated cells. We therefore calculated the *LDi* as the ratio between the standard deviation to the mean and the mean of cone spacing, which is statistically equivalent to the coefficient of variation and provides a measure of the spread in cone neighbour distances.

**Fig 4:**
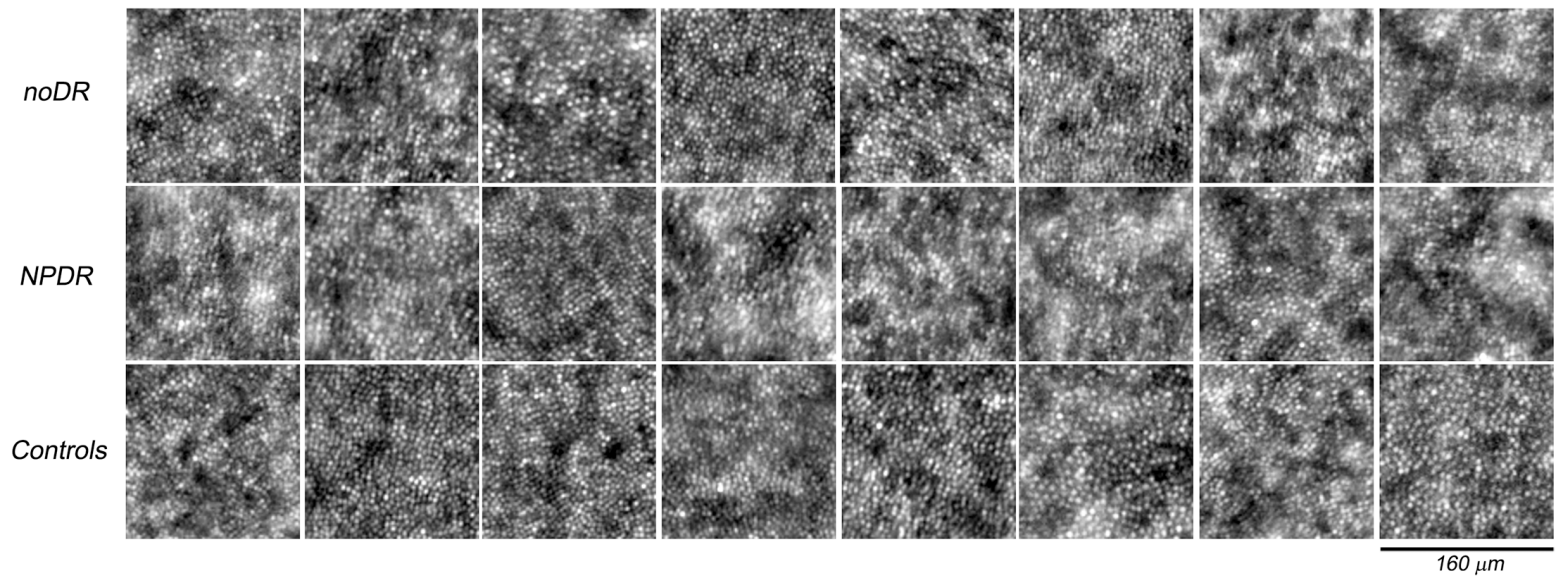
Adaptive optics images of the photoreceptor mosaic for all patients with diabetes and eight representative age-matched controls acquired at 1.50 degrees superior from the fovea. The upper row includes the eyes with noDR (from DM1_P9 to DM1_P16 from the left to right respectively), the middle row the eyes with NPDR (from DM1_P1 to DM1_P8 from the left to right respectively) and the lower row the control eyes (C30, C32, C33, C37, C39, C41, C44 and C48 from the left to right respectively). Each window is 160x160 μm. The cones were reliably identified in all AO images collected from study cases and controls.

Starting from analysis of Voronoi diagrams, the *HPi* provided valuable information on the increase in non hexagonal cones in the parafovea of DM1 patients. Since we found a significantly greater percentage of 4-sided and 8-sided cells in diabetic eyes than controls, we used their fractional increase to statistically describe the deviation of the cone mosaic from normal arrangement. Overall, this index aimed at identifying pathological changes of the intricate non-random arrangement of cones.[8,12,17,25] Previous analysis has demonstrated the presence of lattice defects distributed non-randomly even in the normal human retinal mosaic. Cone positions with non-hexagonal neighbours have been shown to be arranged in linear series subdividing the mosaic in a series of patches of 6*n* cones with varying axial orientation and to form “point defects” of the continuous crystalline cone lattice.[8,9,11,13,26,29] Either the significantly lower percentage of 6*n* or the higher percentage of 4*n* and 8*n* in DM1 patients than controls cannot be explained by the effect of aging, low quality or artefacts in the images of the cone mosaic, as shown in Fig 4. In the present control population (age range; 27–56 years), we did not find any significant age-related differences in percentages of 6*n* (r = -0.07; P = 0.92), 4*n* (r = 0.02; P = 0.26) and 8*n* (r = 0.07; P = 0.16) among subjects; a slight negative correlation was observed between age and density (r = -0.40; P = 0.08), as also found by Park et al.[30] (r = -0.12; P = 0.14), who have evaluated 192 adults with mean age of 33.6±13.2 years. Further evidence of comparable quality between AO images collected from DM1 patients and controls was provided by the accuracy of the algorithm used for identifying the cones in both groups. The results were also consistent with those shown previously using the same sampling area (% of manually added cones: 1.3±0.7%).[10] If necessary, each sampling window was slided (maximum sliding: 0.12 degrees) along the isoeccentric ellipse at 1.5 degrees retinal eccentricity;[31] this approach was done in order to avoid areas of the cone mosaic masked by large vessels or haemorrhages thus removing any bias due to *undersampling effects* between subjects.[8–10]

The cellular and molecular mechanisms that control cellular pattern formation in the adult human retinal mosaic are almost unexplored. A study [11] on an enucleated human eye with previous diagnosis of advanced glaucoma has shown a lower cone peak density and significantly fewer cones with hexagonal Voronoi domains in the fovea as compared to healthy eyes. In other vertebrates, there is evidence that cone mosaic rearrangement follows selective or unselective photoreceptor death.[32] Collectively, these studies are consistent with the understanding that the mechanisms that spatially regulate physiological (due to retinal development and aging) or pathological (due to retinal degeneration) cellular pattern formation are different.[33–37] The present findings reinforce the hypothesis that a rearrangement of the cone mosaic may be caused by pathological decreased cone density in the adult human retina.[35] Under this hypothesis, the pathological changes of the cone mosaic spatial organization found in this population of diabetic adult patients may correspond to common mechanisms of degeneration in other acquired retinal diseases, as for example in type 2 diabetes. In previous study Zagers et al.[38], using a foveal reflection analyser, identified changes in the integrity of the foveal photoreceptors in a cohort of 14 patients (mean age: 46±11 years), showing no differences between patients with type 1 or type 2 diabetes. Nevertheless, more research is needed before firm conclusions can be drawn, including investigation on the pattern of pathological changes of the cone mosaic in study populations with different retinal diseases.

The results from this pilot study support the neurodegenerative theory, for which the retinal neuronal cells, including photoreceptors, are involved early in the course of diabetes in patients.[39–42] Retinal neurodegeneration has been also supposed to participate in the development of microvascular abnormalities.[43,44] No attempt to correlate the AO findings with functional measures of the retina was done in this study, since microperimetry has been shown previously to hold low sensitivity to detect central dysfunction in patients with noDR or mild NPDR and no macular edema.[4]

In conclusion, the adaptive optics retinal imaging biomarkers of cone density, *LDi* and *HPi*, taken together, identified with accuracy the pathological disruption of the parafoveal cone mosaic in patients with type 1 diabetes, even before any sign of diabetic retinopathy was found on fundoscopy. Further analysis on a large cohort of patients would be helpful to understand the potential of AO based imaging biomarkers to enhance screening strategies in patients with diabetes in the near future.

## Supporting Information

S1 Fig Results of the logistic regression model used for discriminating the parafoveal cone mosaic spatial arrangement in patients with type 1 diabetes mellitus and no signs of diabetic retinopathy on fundoscopy from age-matched controls. The combined use of cone density, cone spacing and Voronoi diagrams as descriptors in a logistic regression model achieved 100% accuracy to discriminate the spatial distribution and arrangement of the parafoveal cone mosaic between patients with type 1 diabetes mellitus and no signs of diabetic retinopathy on fundoscopy (noDR cases) and age-matched controls. The symbols 0 and 1 represent controls and noDR cases respectively. Each symbol represents 1.25 cases or controls.
(TIF)
