# Supplementary figures and images for: Investigation of Adaptive Optics Imaging Biomarkers for Detecting Pathological Changes of the Cone Mosaic in Patients with Type 1 Diabetes Mellitus
Source: PLoS One. 2016 Mar 10;11(3):e0151380. doi: 10.1371/journal.pone.0151380 (PMC4786294; doi:10.1371/journal.pone.0151380)

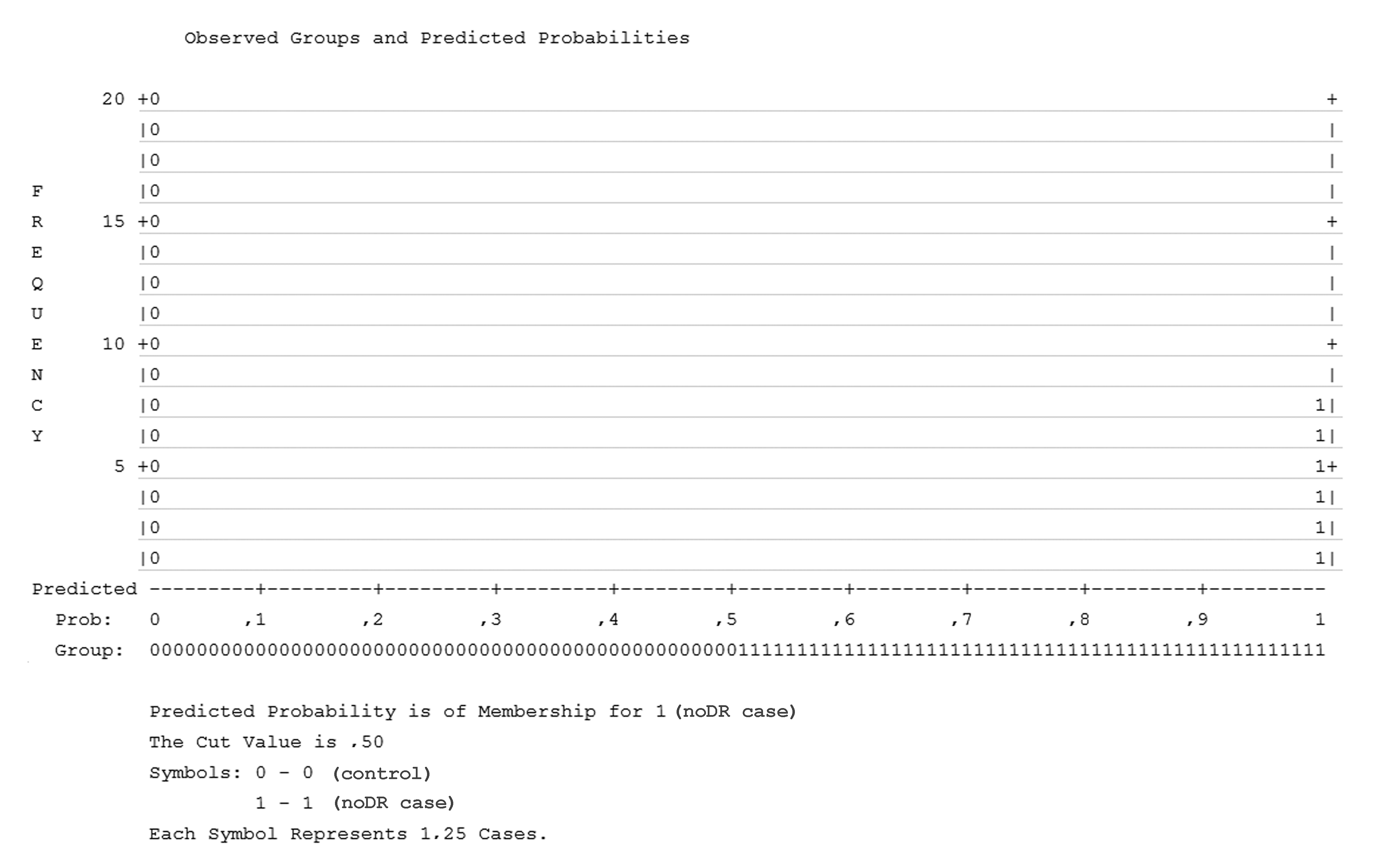

Supplement: S1 Fig — The combined use of cone density, cone spacing and Voronoi diagrams as descriptors in a logistic regression model achieved 100% accuracy to discriminate the spatial distribution and arrangement of the parafoveal cone mosaic between patients with type 1 diabetes mellitus and no signs of diabetic retinopathy on fundoscopy (noDR cases) and age-matched controls. The symbols 0 and 1 represent controls and noDR cases respectively. Each symbol represents 1.25 cases or controls. (TIF) [file pone.0151380.s001.tif]
